# Heme Oxygenase-1 Activity as a Correlate to Exercise-Mediated Amelioration of Cognitive Decline and Neuropathological Alterations in an Aging Rat Model of Dementia

**DOI:** 10.1155/2018/7212861

**Published:** 2018-01-30

**Authors:** Andrea Kurucz, Mariann Bombicz, Rita Kiss, Dániel Priksz, Balázs Varga, Tibor Hortobágyi, György Trencsényi, Renáta Szabó, Anikó Pósa, Rudolf Gesztelyi, Zoltán Szilvássy, Béla Juhász

**Affiliations:** ^1^Department of Pharmacology and Pharmacotherapy, University of Debrecen, Debrecen, Hungary; ^2^MTA-DE Cerebrovascular and Neurodegenerative Research Group, Department of Neurology & Neuropathology, University of Debrecen, Debrecen, Hungary; ^3^Department of Nuclear Medicine, University of Debrecen, Debrecen, Hungary; ^4^Department of Physiology, Anatomy and Neuroscience, University of Szeged, Szeged, Hungary

## Abstract

Alzheimer's disease (AD) is a neurodegenerative disorder with cognitive impairment. Physical exercise has long been proven to be beneficial in the disorder. The present study was designed to examine the effect of voluntary exercise on spatial memory, imaging, and pathological abnormalities. Particular focus has been given to the role of heme oxygenase-1 (HO-1)—an important cellular cytoprotectant in preserving mental acuity—using an aging rat model of dementia. Male and female Wistar rats were segregated into six groups—namely, (i) aged sedentary (control) females (ASF, *n* = 8); (ii) aged sedentary (control) males (ASM, *n* = 8); (iii) aged running females (ARF, *n* = 8); (iv) aged running males (ARM, *n* = 8); (v) young control females (YCF, *n* = 8); and (vi) young control males (YCM, *n* = 8). Rats in the ARF and ARM groups had free access to a standardized inbuilt running wheel during the 3-month evaluation period. Spatial memory was investigated using the Morris Water Test, imaging and pathological alterations were assessed using positron emission tomography (PET) imaging and histopathological examinations (H&E, Congo red staining), respectively, and HO-1 enzyme activity assays were also conducted. The outcomes suggest that voluntary physical exercise mitigates impaired spatial memory and neuropathological changes exhibited by the aging sedentary group, via elevated HO-1 activity, contributing to the antioxidant capacity in the aging brain.

## 1. Introduction

Alzheimer's disease (AD) is a progressive neurodegenerative disorder, with neuropathology characterized by the accumulation of extracellular amyloid plaques and intracellular neurofibrillary tangles. In 2016, Alzheimer's Disease International, an organization founded to promote efforts to prevent and treat the disease, issued their World Alzheimer's Report, which estimates that 46.8 million people worldwide were living with AD, with 131.5 million projected to be afflicted by 2050 [1]. Changes in demographic patterns, principally increasing life expectancy, with resulting increases in elderly populations, have resulted in a concomitant expansion of AD-afflicted individuals and an increased burden on national healthcare systems. According to Alzheimer's Association, AD is the 6th leading cause of death in the USA, with 1 out of every 3 elderly people succumbing to it in 2016. In that year, the estimated fiscal cost of the disease was 236 billion dollars, which is forecast to increase to a trillion dollars annually by 2050. These factors notwithstanding, the development of effective countermeasures for AD remains elusive at the time of this writing [1].

Although comprehensively definitive descriptions of AD pathomechanisms remain undefined, some major contributing factors have been elucidated. For example, evidence of a signaling cascade triggered by amyloid deposits, resulting in neuroinflammation and microtubule-associated tau protein hyperphosphorylation, is a likely major underlying process contributing to AD pathogenesis [2]. An element of this phenomenon is amyloid precursor protein (APP), a transmembrane component of the outer membrane of the cell. Also, the mitochondria is cleaved by three enzymes *α*-, *β*-, and *γ*-secretases, with variable cleavage outcomes, determined by as-yet undefined factors. Exploration of this enzymatic activity reveals that cleavage by *α*-secretase prevents amyloid *β*-formation—and the main products of cleavage by *β*- and *γ*-secretases include A*β*40 and A*β*42, which are secreted extracellularly, and may form aggregations that contribute to neurological tissue damage [3–5].

Tau protein aggregation is promoted by amyloid *β*, which may directly or indirectly activate protein kinases, such as GSK3 and CDK5, via elevation of intracellular calcium levels, leading to tau-hyperphosphorylation and aggregate formation [6, 7]. Once tau aggregation is initiated, the resulting cascade becomes self-sustaining, continuing independently of amyloid *β* presence [2]. Rogers et al. described how amyloid *β* and its N-terminal fragments bind to C1q, thus directly activating the complement system and downstream neuroinflammatory processes that play roles in evolution of the disease [8]. As a result of these events, microglial and astrocytic activation of the kinase/phosphatase activity can be altered, resulting in further hyperphosphorylation and tau protein aggregation [9].

Several studies have demonstrated that oxidative stress, mitochondrial dysfunction, and inappropriately high proinflammatory activity synergize to damage macromolecules on which healthy cellular and tissue function are dependent—including nucleic acids, lipids, and proteins, ultimately causing neurodegeneration [10–12].

Ongoing characterization of AD and other neurodegenerative disorders have revealed that therapeutic manipulation of the heme oxygenase/biliverdin reductase (HO/BVR) system holds enormous potential for prevention and remediation of these diseases. HO is a major cellular cytoprotectant, the upregulation of which is an early event of the adaptive response to stress [13]. In humans, rodents, and many other species, HO exists as two main isoforms: HO-1, an inducible form, and HO-2, which is a constitutively active form. Both isotypes catalyze degradation of heme into biliverdin-alpha (BV-alpha), carbon-monoxide (CO), and ferrous ion [Fe(II)]. HO-1, also known as heat shock protein-32 (Hsp-32) is expressed at increased levels in response to internal or exogenous stress cues, including the following: oxidative stress, ischemia, reperfusion, thermal effects, bacterial lipopolysaccharide, and the presence of hemin, its main substrate, which accumulates in tissues as a result of red blood cell turnover [14]. HO-2 expression is observed to increase in response to developmental factors, adrenal glucocorticoids, and nitric oxide (NO) [15, 16]. The gene coding for HO-1,* HMOX1*, has two upstream enhancer regions—E1 and E2—which contain antioxidant-responsive elements supporting the oxidative-inducible nature of this protein [17]. Maines suggested that HO-1 plays a pivotal role in the earliest phases of homeostatic adaptive responses that have evolved to limit tissue damage, while HO-2 is more likely to act as a housekeeping molecule in ways that contribute to the maintenance of heme homeostasis [18]. CO, which is a major metabolite resulting from HO-mediated degradation of heme, regulates important mechanisms in the central nervous system. These include long-term hippocampal potentiation and neurotransmitter release. HO also functions in the peripheral nervous system to catalyze a diverse range of vital activities, such as the following: nonadrenergic, noncholinergic gastrointestinal relaxation; vasodilation; and inhibition of platelet aggregation. Moreover, the enzyme acts to limit apoptotic depletion of healthy cells in response to oxidative stressors, along with other cytoprotective functions [19–23].

HO-mediated heme degradation produces biliverdin, which is almost immediately reduced to bilirubin (BR) by biliverdin reductase (BVR). Two BVR isoforms have been described: BVR-A and BVR-B. However, only BVR-A reduces BV-alpha into BR, which is a strong antioxidant and antinitrosative molecule [24]. Several investigations have demonstrated that accumulation of amyloid *β* acts to increase HO-1, in a physiologic regulatory feedback process which ameliorates the harmful effects of amyloid *β* [25, 26]. Additionally, many studies which describe the protective role of physical activity as a countermeasure to AD-related neurodegenerative processes include underlying cellular and molecular mechanisms in which HO activity is a potential factor for humans and in animal models of AD [27]. The signaling events linking physical activity with reduction in severity of AD include activity of the Wnt pathway, along with influences that suppress aberrant neuronal apoptosis or decrease amyloid *β* accumulation through its enzymatic degradation and improvement of synaptic plasticity and neurogenesis [28–30], or by increasing the expression of sirtuin-1/peroxisome proliferator-activated receptor gamma coactivator 1-alpha (SIRT-1/PGC-1*α*) signaling, therefore inhibiting amyloid *β* production [31].

Insight into the above events is likely to yield improved understanding to AD etiology which, at the time of this writing, remains substantially obscure. Most cases of AD occur spontaneously, with triggering events that are poorly defined or unknown. Familial susceptibility to the disorder occurs, but it is definitively implicated in only 1–5% cases [32]. Several animal models have proven to be suitable investigative tools for research into AD pathogenesis. The most widely used animals for modeling AD are mice and rats. Due to the lack of complete understanding of AD pathomechanisms, all of these models have significant limitations. However, careful selection of a particular animal model in the context of a particular AD-related hypothesis to be tested may reduce the influence of confounding factors associated with a particular model. For example, the numerous transgenic mouse models exhibit features that make them particularly useful in characterizing the familial form of AD, while healthy animals that are subjected to treatments such as streptozotocin- (STZ-) induced neurodegeneration may be used to demonstrate phenotypic characteristics of the spontaneously occurring forms of AD [33]. The authors of the present investigation selected a model in which neurodegeneration occurred naturally, though advanced age (18-month-old rats), thus avoiding the potentially confounding influences of transgenes or drugs.

The present investigation was structured to test a hypothesis that voluntary (recreational) physical activity (treadmill exercise) ameliorates neurodegeneration-related cognitive decline and associated major neuropathological alterations by decreasing oxidative stress via increasing HO-1 activity, thereby preventing neuronal loss in the brain of aging rats. This is the first study to evaluate this possibility using an animal model as a correlation between physical exercise and molecular-biological effects associated with age-related neurodegeneration.

## 2. Materials and Methods

### 2.1. Animals

18-month-old male and female Wistar rats, weighing 300–350 grams at the outset of each experiment, were housed two per cage at an ambient temperature of 22–24°C under a 12 h : 12 h light : dark cycle with food and water ad libitum.

### 2.2. Treatment Group Assignment

Animals used in the present study were segregated into six groups, defined as follows: (i) aged sedentary female rats (ASF, *n* = 8); (ii) aged sedentary male rats (ASM, *n* = 8); (iii) aged running female rats (ARF, *n* = 8); (iv) aged running male rats (ARM, *n* = 8); (v) young (3 months old) control female rats (YCF, *n* = 8); and (vi) young (3 months old) control males (YCM, *n* = 8). Rats in the ARF and ARM groups had free availability to a standardized inbuilt running wheel and were able to exercise freely, whereas animals in the ASF and ASM groups were housed without access to running wheels. All groups were maintained in the above described conditions for 3-month periods.

### 2.3. Behavioral Tests

Here, the Morris Water Maze (MWM) test was used as a primary measure of memory function by animal subjects. MWM data is here considered to be particularly valuable, since it is one of the most frequently used learning and spatial memory assessments for rodent models, and it is acknowledged to produce reliably reproducible outcomes [34]. MWM evaluations were conducted as follows: a circular pool, 150 cm in diameter, 50 cm in height, in a small quiet room, is filled with opaque water at room temperature, to a depth of 30 cm, and divided into four quadrants. An invisible platform, 10 cm in diameter, is submerged 1 cm below the surface, in the middle of one of the four quadrants. The position of the platform is kept unaltered throughout a training session, during which time each animal becomes familiar with structural features and visual cues, memories of which are the basis of the test. Selected visual cues are placed on the inner wall of the pool to indicate the four quadrants and provide navigation reference points, which the rats will remember to varying degrees, depending on their neurological capacity. MWM tests were carried out during three consecutive days, twice a day before the treatment (baseline), and after the 3 months of voluntary running on wheels installed in cages. For each trial, the animals were gently put into the water at one of the four starting points (that differed for each test—the sequences for which were selected randomly). The tests were scored on the basis of ability of the animals to locate the submerged platform. Each animal was allowed a 10-second rest period on the platform. If an animal was unable to locate a platform within a 60-second interval, it was gently guided and placed on it. Escape latency time and swimming patterns were measured by a video tracking system (EthoVision 2002, Noldus Information Technology, Netherlands).

### 2.4. Small Animal PET Imaging Using Radiopharmaceuticals

Rats were injected with 10.0 ± 0.2 MBq of [^11^C]PIB via the lateral tail vein. 30 minutes following injection of the radiotracer, the animals were anaesthetized using 3% isoflurane with a dedicated small animal anesthesia device. Next, 15-minute static single-frame PET scans were acquired using a small animal PET scanner (MiniPET-II) to visualize the brain. Scanner normalization and random correction were applied on the data, and the images were reconstructed with the standard EM iterative algorithm. The pixel size was 0.5 × 0.5 × 0.5 mm, and the spatial resolution varied between 1.4 to 2.1 mm from central to 25 mm radial distances. The system sensitivity is 11.4% [35].

#### 2.4.1. PET Data Analysis

Radiotracer uptake was expressed in terms of standardized uptake values (SUVs). Ellipsoidal 3-dimensional volumes of interest (VOI) were manually drawn around the edge of the brain activity by visual inspection using BrainCad software. The standardized uptake value (SUV) was calculated as follows: SUV = [VOI activity (Bq/ml)]/[injected activity (Bq)/animal weight (g)], assuming a density of 1 g/cm^3^.

### 2.5. Histology Studies: Hematoxylin-Eosin and Congo Red Staining

Following the MWM test at the end of the three-month recreational exercise, the rats were deeply anesthetised and transcardially perfused with 10 ml of 4°C PBS, followed by 30 ml of 4°C paraformaldehyde solution (4% in phosphate buffer, pH 7.4). Rat brains were removed and bisected into two halves. The right hippocampal, frontal, and temporal lobes were removed and postfixed for 24 hours in the same fixative (4°C) and subsequently placed in a 30% sucrose solution for 72 hours at 4°C in PBS. Formalin-fixed, paraffin-embedded brain tissue blocks (FFPE) were then microtomed into 7 *μ*m thick sections and stained with hematoxylin-eosin (H&E) and Congo red (Sigma-Aldrich) for detection of amyloid-related pathology. The sections were placed on slides, dried at 37°C overnight, then rehydrated using a graded alcohol series, and then stained with hematoxylin for 3 minutes. Sections were rinsed in running tap water for 10 minutes until they turned blue and then stained with eosin for 3 minutes. Congo red-stained sections were subsequently counterstained with hematoxylin for 3 minutes, rinsed in running tap water to develop blue coloration, washed twice in distilled water, and stained with Congo red for 40 minutes. The stained sections were subsequently analyzed by light microscopy. Examination of Congo red-stained tissues was conducted using polarized light. Semiquantitative analysis of amyloid pathology was accomplished by first counting the number of amyloid plaques and Congo red-positive vessels as a fraction of the total number of vessels observed in 10 fields, using a 10x objective (100x magnification). The resulting percentage of Congo red-positive vessels within the total number of vessels (number of positive vessels/total vessel number × 100) yielded quantitative data that allowed assignment of tissues into 4 different categories, described as follows: (i) tissue with no (0%) positive vessels (-); (ii) tissue with 1–29% (low number of positive vessels) (+); (iii) tissue with 30–69% (moderate number of positive vessels) (++); and (iv) tissue with more than 70% (numerous positive vessels) (+++).

### 2.6. Immunohistochemical Study

Immunohistochemistry (IHC) has been performed according to standardized methods. In brief, 7 *μ*m thick sections from formalin-fixed, paraffin-embedded blocks have been stained for anti-beta amyloid 1–42 antibody (Abcam, 1 : 200) according to the manufacturer's protocol. The stained sections were analyzed by light microscopy. Semiquantitative analysis of amyloid pathology was accomplished by a similar method to that of the Congo red staining. We counted the number of anti-beta amyloid 1–42 antibody-positive vessels as a fraction of the total number of vessels observed in 10 fields, using a 10x objective (100x magnification).

The resulting percentage of positive vessels within the total number of vessels (number of positive vessels/total vessel number × 100) yielded quantitative data that allowed assignment of tissues into 4 different categories, described as follows: (i) tissue with no (0%) positive vessels (-); (ii) tissue with 1–29% (low number of positive vessels) (+); (iii) tissue with 30–69% (moderate number of positive vessels) (++); and (iv) tissue with more than 70% (numerous positive vessels) (+++).

### 2.7. Measurement of Heme Oxygenase Activity

A widely used and reliable assay for HO-1 activity, based on the reduction of biliverdin into bilirubin, was used for the present study [36]. Following the sacrifice of each animal, the frontal cortex, temporal cortex, and hippocampus of harvested brains were isolated and homogenised (Ultraturrax T25; 13,500/s; 2 × 20 s). Samples from the left hemispheres of each brain were processed using 10 mM* N*-[2-hydroxyethyl]piperazine-*N*′-[2-ethanesulfonic acid] (HEPES), 32 mM sucrose, 1 mM dithiothreitol (DTT), 0.1 mM EDTA, 10 *μ*g/ml soybean trypsin inhibitor, 10 *μ*g/ml leupeptin, and 2 *μ*g/ml aprotinin, pH 7.4. The supernatant was collected by centrifugation for 30 minutes at 20,000 ×g at 4°C. Each reaction mixture contained the following in a final volume of 1.5 ml : 2 mM glucose 6-phosphate, 0.14 U/ml glucose 6-phosphate dehydrogenase, 15 *μ*M heme, and 150 *μ*M *β*-nicotinamide adenine dinucleotide phosphate (NADPH). 120 *μ*g/ml rat liver cytosol was used as a source of biliverdin reductase, with 2 mM MgCl_2_, 100 mM potassium phosphate buffer, and 150 *μ*l of supernatant. Incubation was carried out in the dark at 37°C for 60 minutes. The reaction was stopped by putting samples on ice. The bilirubin formed was calculated from the difference between optical densities obtained at 460 nm and 530 nm. One unit of heme oxygenase activity was defined as the amount of bilirubin (nmol) produced per hour per mg of protein.

### 2.8. Graphs and Statistical Analysis

For statistical analysis and figure plotting, GraphPad Prism 7.03 Software (GraphPad Prism Software Inc., California, USA) was used. All data were expressed as means ± SEM. Two data sets were compared with unpaired Student *t*-test or *t*-test with Welch's correction (if equal variance test was not passed). More than two data sets were compared with one-way ANOVA (using Geisser-Greenhouse correction) with Tukey post hoc testing. (All data sets passed the D'Agostino-Pearson omnibus normality test.)

## 3. Results

### 3.1. Voluntary Exercise Improves Spatial Learning and Memory in the Aging Model of Alzheimer's Disease

**Figure 1:**
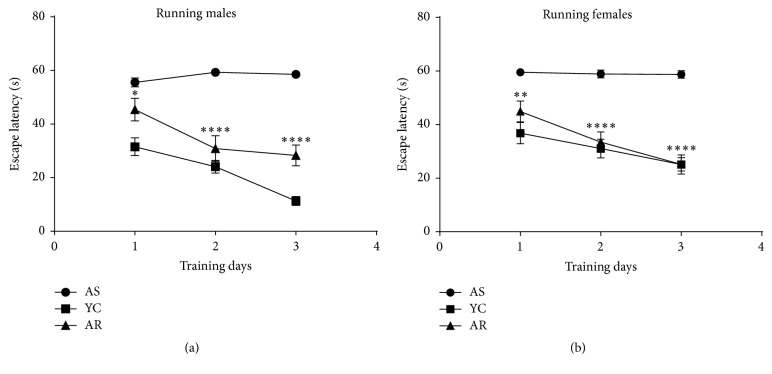
*Effect of recreational exercise on Morris Water Maze performance.* AS, aged sedentary; AR, aged running; YC, young control. *∗* means *p* < 0.05 compared to the control, *∗∗* means *p* < 0.01 compared to the control, and *∗∗∗∗* means *p* < 0.001 compared to the control.

Following 3 months of voluntary exercise, rats were assessed for spatial learning and memory using the Morris Water Maze test. Assessments based on swim time to find submerged platforms (escape latency) were followed with a tracking system. Escape latency scores revealed a significant correlation between voluntary exercises on the three training days of the MWM test protocol (Figure 1). Animals within the aged sedentary group (including both male and female rats) required significantly more time to find the platform and were not able to improve their learning ability (*p* < 0.001). However, animals in the aged running groups (both males and females) were more likely to find the platform within decreasing time compared to the sedentary aging groups (mean differences: Day 1: 10.15 seconds, *p* = 0.038, and 14.60, *p* < 0.01; Day 2: 28.45, *p* < 0.001, and 25.45, *p* < 0.001; Day 3: 30.25, *p* < 0.001, and 33.55, *p* < 0.001 for males and females, respectively.

### 3.2. ^11^C-Pittsburgh Compound-B PET Region of Interest (ROI) Analysis in Amyloid Imaging

**Figure 2:**
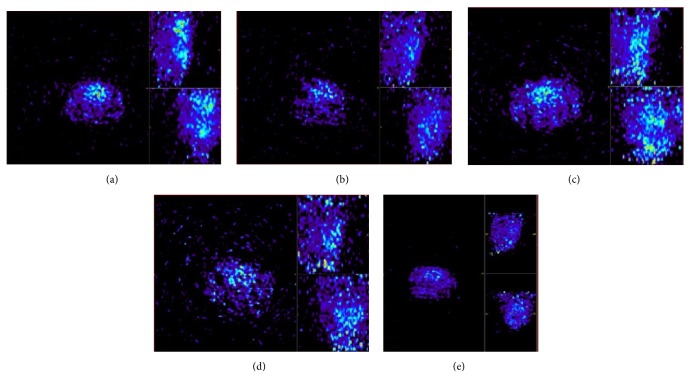
PET images (one coronal, two horizontal sections in each picture) of aged sedentary female (ASF) and male (ASM) rats (a, c), aged running female (ARF) and male (ARM) rats (b, d), and a young control animal (e).

**Figure 3:**
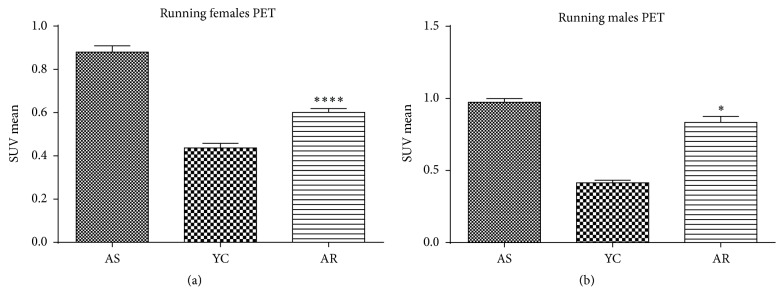
*Effect of recreational exercise on PIB retention. (a) Aged males and (b) aged females.* AS, aged sedentary; AR, aged running; YC, young control. *∗* means *p* < 0.05 compared to the control and *∗∗∗∗* means *p* < 0.001 compared to the control.

Figure 2 shows PET scan images of rat brains belonging to each test group used. PIB radionuclide retention was significantly higher among aged sedentary animals than among young control rats (SUV mean of PIB for aging females: 0.87 ± 0.029 and for young control females: 0.43 ± 0.02).  Figure 3(a) shows data for aged males: 0.97 ± 0.024 and for young control males: 0.41 ± 0.01 (Figure 3(b): *p* < 0.001 for both males and females). Analysis of data from animals in the aged running groups revealed significantly lower PIB retention (*p* = 0.0119 and *p* < 0.001, for males and females, resp.) than in the young groups.

It is nevertheless important to caveat the above results with a corollary observation: that rat brain PIB retention of aged running male and female rats showed no significant correlation with the spatial learning and memory exhibited by these animals. Aged male and female running rats performed equally well in the Morris Water Maze test.

### 3.3. Voluntary Exercise Ameliorated the Neurodegenerative Histopathological Changes in the Brains of Aging Rats

**Figure 4:**
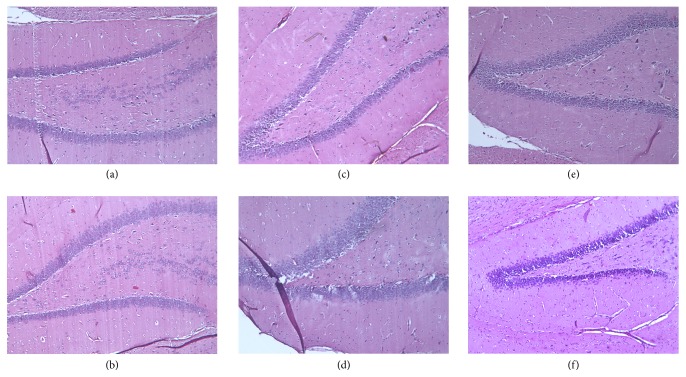
*Hematoxylin-eosin stained sections (100x magnification).* (a-b) Hippocampus of a young control male and a female animal, respectively. (c-d) Hippocampus of an aged sedentary male and a female rat, respectively. (e-f) Hippocampus of an aged running male and a female animal, respectively.

**Figure 5:**
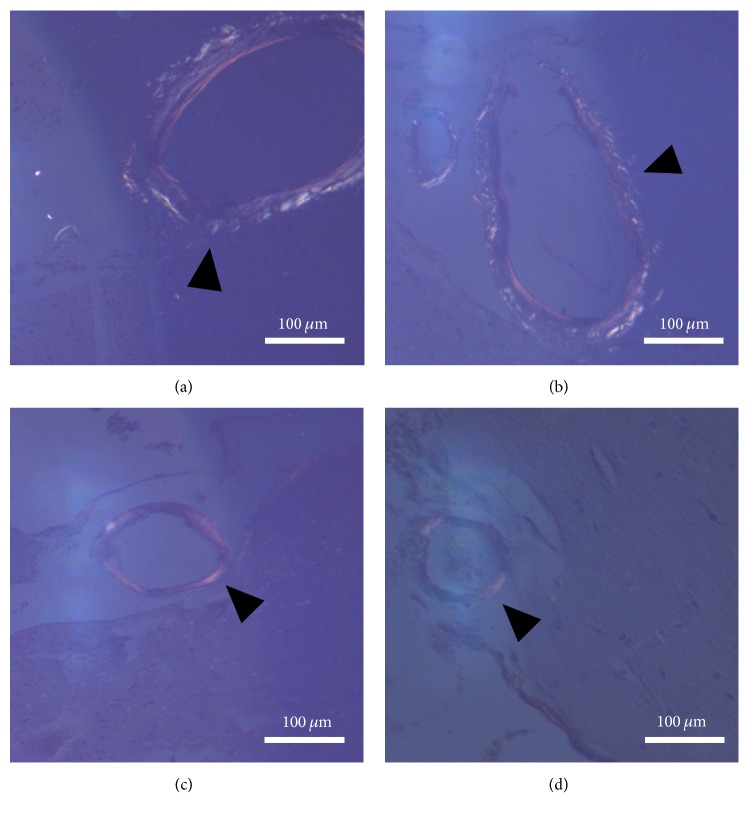
*Cerebral amyloid angiopathy represented by Congo red staining (100x magnification).* (a-b) Aged sedentary male and female brain sections under polarized light. The bright regions in the vessel walls marked by the black arrowheads can be identical to amyloid-like depositions. (c-d) Aged running male and female brain sections under polarized light. The black arrowheads are pointing to vessels containing lesser amount of amyloid-like depositions.

**Figure 6:**
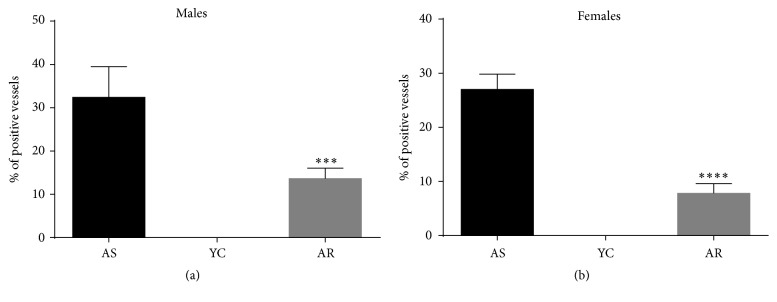
*Statistical analysis of CAA.* ^*∗∗∗*^ *p* < 0.001; ^*∗∗∗∗*^*p* < 0.001.

**Figure 7:**
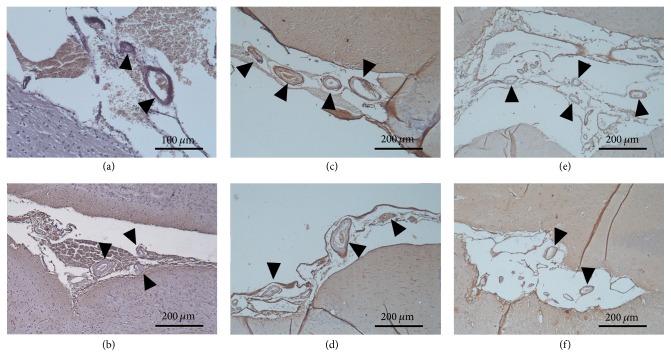
*Cerebral amyloid angiopathy represented by immunohistochemistry (100x magnification).* (a-b) Brain section from a young control male and a female animal, respectively. (c-d) Brain section from an aged sedentary male and a female rat, respectively. (e-f) Brain section from an aged running male and a female animal, respectively. The brownish color represents the amyloid-like depositions in the vessel walls.

**Figure 8:**
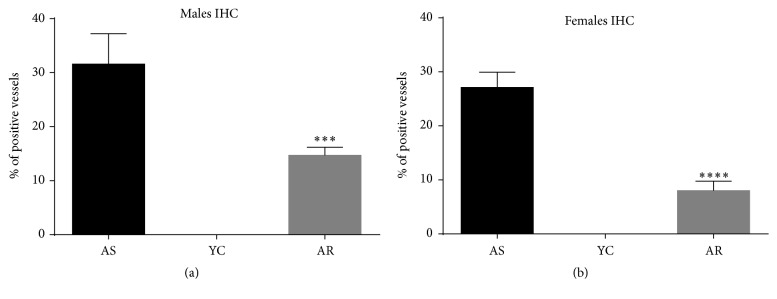
*Statistical analysis of CAA immunohistochemistry.* ^*∗∗∗*^ *p* < 0.001; ^*∗∗∗∗*^*p* < 0.001.

The sagittal sections of aged sedentary (AS) and aged running (AR) animals were examined by light microscopy, using H&E and Congo red staining. H&E staining revealed remarkable degenerative abnormalities in the AS groups, compared to the young control animals (see Figures 4(a) and 4(b)). Disintegration of the pyramidal layer structure, neuronal loss, and severe pericellular edema was observed in the dentate gyrus (see Figures 4(c) and 4(d)). However, following the 3 months of voluntary exercise, these changes were attenuated to some extent. Cellular substructure of tissues from rats allowed exercising displayed significantly tighter organization, along with lower levels of pericellular edema (Figures 4(e) and 4(f)). The accumulation of amyloid in rat brains demonstrated by Congo red staining failed to demonstrate amyloid plaques. However, age-related cerebral amyloid angiopathy (CAA) was detectable, as revealed in Figure 5. The aged sedentary animals (AS)—both females and males—exhibited a moderate number of Congo red-positive vessels (32.4 ± 2.237% in males and 27 ± 0.89% in females). By contrast, tissues from animals in the running groups possessed significantly lower density of Congo red-positive vessels (13.60 ± 0.77% in males and 7.8 ± 0.57% in females) and their Congo positivity was less intense. Statistical analysis of the results proved that the difference between the aged sedentary and aged running groups was significant both in the case of males (*p* < 0.001) and in the case of females (*p* < 0.001) (Figure 6.). Age-related cerebral amyloid angiopathy (CAA) was also detectable by immunohistochemistry (Figure 7.). The aged sedentary animals (AS)—both females and males—exhibited a moderate number of Congo red-positive vessels (31.6 ± 1,78% in males and 27,1 ± 0.9% in females). However, tissues from the running aging groups possessed significantly lower density of anti-beta amyloid 1–42 antibody-positive vessels (14.70 ± 0.47% in males and 8 ± 0.55% in females). The difference between the aged sedentary and aged running groups was statistically significant both in the case of males and in the case of females (Figure 8).

### 3.4. Effects of Voluntary Exercise on HO-1 Activity

**Figure 9:**
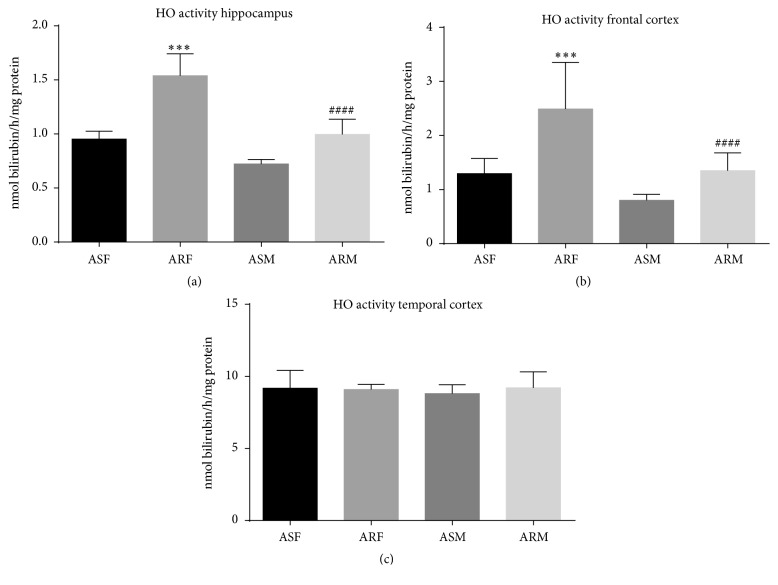
*Heme oxygenase-1 enzyme activities of the indicated experimental groups categorized by brain regions.* ASF, aged sedentary females; ARF, aged running females; ASM, aged sedentary males; ARM, aged running males. ^*∗∗∗*^*p* < 0.001; ^####^*p* < 0.001.

Relatively low activity of the heme oxygenase-1 enzyme was observed in the frontal cortex and hippocampus of aged sedentary rats both in females (1.3 ± 0.08 and 0.95 ± 0.02 nmol/h/mg protein, resp.) and in males (0.8 ± 0.03 and 0.72 ± 0.04 nmol/h/mg protein, resp.). Interestingly, three months of voluntary exercise correlated significantly with increased activity of this enzyme, both in females (2.49 ± 0.08, *p* < 0.001, and 1.53 ± 0.06 nmol/h/mg protein, *p* < 0.001, resp.) and in males (1.35 ± 0.1, *p* < 0.001, and 0.99 ± 0.04 nmol/h/mg protein *p* < 0.001, resp.). Moreover, within the temporal cortex, excluding the hippocampus, higher HO-1 activity was noted both in females (9.18 ± 0.39 nmol/h/mg protein) and in males (8.80 ± 0.19 nmol/h/mg protein). Nevertheless, no correlation was observed between HO-1 activity and exercise status for the above conditions either in females (9.08 ± 0.11 nmol/h/mg protein) or in males (9.2 ± 0.34 nmol/h/mg protein) (Figure 9.).

## 4. Discussion

Alzheimer's disease (AD) is a chronic neurodegenerative disorder that is the most common cause of dementia in the elderly, and it is clinically characterized by progressive loss of cognitive ability, including memory, communication, judgement, and reasoning. Features of the disorder include mood swings, personality disorders, and progressively adverse behavior. During the late stages of the disease, afflicted individuals may become unable to care for themselves [37]. The underlying pathomechanisms of this tragic syndrome appear to include accumulation of amyloid-*β* oligomers and aggregation of this material into plaques, which disrupt the metabolism of affected cells in ways that promote neuroinflammation. These processes trigger hyperphosphorylation of the tau protein, which normally plays a role in microtubule stabilization, but, with AD-associated hyperphosphorylation, it mediates synaptic dysfunction and cell death [38].

Numerous animal models, such as drug-treated rodents (e.g., STZ-treated rats) and various transgenic animals (e.g., APP (swe)/PS1(e9d)1 transgenic mouse strain, Tg2576 mouse model) [39, 40], have been developed to parallel the pathogenesis of human Alzheimer's disease in ways that allow their use in creation of novel preventive and therapeutic strategies.

Nevertheless, optimal utilization of such animals is currently constrained by lack of clear, mechanistic insight into AD pathomechanisms [33]. Moreover, most human AD cases occur spontaneously, without clearly defined etiologic contributors. Indeed, only 5% of these patients are positively diagnosed with the familial form of the disease. Thus, the present study examined neurodegenerative changes in an aging rat model, in which the animals underwent deterioration of cognitive functions that have significant commonality with AD but occur naturally and thus have better relevance to clinical aspects of spontaneously occurring human AD than drug-induced and transgenic models. The precedent for use of the aging rat as an investigative tool for AD is well established. For instance, Sapronov et al. demonstrated that chronic combined administration of 8-OH-DPAT, a serotonin 5HT (1A) receptor agonist, with galantamine, resulted in pronounced antidepressant and anxiolytic action in an aging rat model of Alzheimer's disease [41]. Moreover, Gocmez et al. used this model to characterize the role of neuroinflammation and reactive oxygen species (ROS) in the pathogenesis of Alzheimer's-type dementia and the effect of resveratrol in decreasing neuroinflammation and preserving cognitive functions of aging rats [42].

A major result of the present study was an observation that elderly animals manifest typical features of dementia. This conclusion was made via use of assays for spatial and learning memory, by direct examination of PET scan images, or histopathological analysis of brain tissue. For example, it was observed that aged rats (both males and females) exhibit Morris Water Maze (MWM) test performance scores significantly worse than those of young rats.

PET scans utilized here revealed significantly high accumulation of amyloid beta in the brains of elderly rats, and histopathological examination of their brain tissue provided novel insight into the extent of neurodegenerative lesions. This included disintegration of the pyramidal layer structure, neuronal loss, and severe pericellular edema in the dentate gyrus, as well as cerebral amyloid angiopathy (CAA). The present study additionally revealed age-related decrease in HO-1 activity within the frontal and parietal cortex and hippocampus.

The above results notwithstanding—and despite significant accumulation of radiolabeled PIB during PET imaging—amyloid plaques were undetectable in the brain tissues by histopathological examination. One explanation for this result might be that PIB cannot differentiate between extracellular amyloid plaques and aggregated amyloid-*β* accumulated in the vessel walls. This conclusion is, nevertheless, speculative and is the subject of ongoing analysis of AD by authors of this report.

Physical exercise ameliorated the cognitive decline in the aged rats. The animals in the aged running (AR) group required significantly shorter escape latency time to remember locations of hidden platforms in the Morris Water Maze test than the aged sedentary (AS) rats, irrespective of their gender. This finding is supported by other recent studies. For example, Yosefi et al. demonstrated that 4-week treadmill running improved the spatial learning and memory of rats with icv, STZ-induced cognitive impairment [43]. Hoveida et al. also demonstrated that daily treadmill exercise for 60 days attenuated cognitive decline caused by bilateral lesions in the nucleus basalis Meynert, which represents another model of Alzheimer's disease [44].

Experiments conducted in this study showed that recreational exercise significantly decreased PIB retention in the brain of aged running animals, in comparison to aged sedentary animals. This outcome suggests that brains of animals that were allowed to exercise accumulated lower amounts of insoluble amyloid-*β* than did sedentary animals. Daily running also attenuated deleterious neuropathological alterations found in the brain tissues of aged rats. Disintegration of the pyramidal layer structure, neuronal loss, and severe pericellular edema in the dentate gyrus of the hippocampus, as well as cerebral amyloid angiopathy (CAA)—represented by Congo red staining and immunohistochemistry—were significantly mitigated by physical activity. The effect of delaying neurodegeneration or restoring synaptic function and neuronal integrity has previously been attributed to physical exercise by numerous studies. For example, Lin et al. reported that regular physical activity restores hippocampal- and amygdala-associated memory and dendritic arbor, along with reducing the severity of edema and neuronal loss [45]. Further, Li et al. demonstrated that moderate exercise improved cognitive function, decreased neuronal apoptosis, and increased synapse numbers in a D-galactose-induced aging mouse model [46].

Oxidative stress, mitochondrial dysfunction, and hyperinflammatory processes constitute a positive feedback process that may be described as a vicious circle, which increases the extent of amyloidogenesis and tau-hyperphosphorylation, leading to neuronal impairment, cell death, and neurodegeneration. This phenomenon has been described by several previous studies to be a major feature of Alzheimer's disease [47–50]. Recent investigations also suggest that HO-1 activity is one of the most important adaptive physiologic countermeasures to AD-associated oxidative stress [51, 52]. Production and biological activation of HO-1 may be induced by a wide range of external and endogenous influences, including oxidative stress, ischemia, heat shock, bacterial lipopolysaccharide, and the presence of hemin, which is the main substrate of heme oxygenases. This enzyme is the rate-limiting macromolecule in heme catabolism and also plays important roles in cell respiration, differentiation processes, energy production, and oxidative biotransformation [14].

For these reasons, natural or pharmacological induction of HO-1 offers enormous promise for prevention and management of many serious chronic diseases, including AD [53, 54]. Many recent studies support this broad objective. For example, Bhardwaj et al. showed that pharmacological induction of HO-1 by hemin decreased oxidative stress and restored cognitive function in an intracerebroventricular streptozotocin-infused rat model of Alzheimer's disease [55]. The authors of the present report conducted the study on which it is based, in part to further demonstrate the future potential of HO-1-based strategies for dealing with the technical challenges posed by AD. Here, the investigators demonstrated significantly increased HO-1 enzyme activity levels in the frontal cortex and hippocampus of aged running rats. Together with the attenuation of cognitive decline, imaging, and neuropathological alterations, the results of these experiments suggest that the severity of age-related Alzheimer's-type dementia may be mitigated by augmentation of naturally occurring HO-1 activity, with outcomes expected to include significantly reduced oxidative stress and reduced severity of AD symptoms.

It has long been known that physical exercise confers many beneficial effects on health. Studies demonstrate that exercise improves cardiovascular function [56], cognitive ability [27], and insulin resistance and symptoms of metabolic syndrome [57] and decreases the severity of neuropsychiatric problems, including depression [58]. Di Loreto et al. demonstrated that regular physical activity potently augmented neuroprotective functions as a correlate of age-related amyloidogenesis and additionally preserved synaptic function [59]. In related studies, Maesako et al. showed that sustained voluntary exercise partly counteracted the effects of a high-fat diet-induced cognitive impairment in APP-overexpressing, transgenic mice. Interestingly, this effect was revealed to occur not by decreasing amyloidogenesis but by increasing the activity of an amyloid-*β* metabolizing enzyme, neprilysin [60]. The present investigation uses the above-mentioned reports as a framework for a comprehensive effort by the authors of this report to develop HO-1 inducers as a mainstay of prevention and therapy of serious chronic disease. Moreover, at the time of this writing, this investigation is the first ever to examine mechanisms of association between physical activity and HO-1 effects. The outcomes shown here demonstrate that HO-1 activity is significantly increased as a result of voluntary running in aging rats and thus has potential for future strategies to treat cognitive decline in AD and related disorders.

## 5. Conclusion

Outcomes of the present study suggest that voluntary running might increase cerebral HO-1 activity at levels that may mitigate oxidative stress present in the brain of rats with age-related Alzheimer's-type dementia.
